# Assessment of Bacterial Accumulation and Environmental Factors in Sentinel Oysters and Estuarine Water Quality from the Phang Nga Estuary Area in Thailand

**DOI:** 10.3390/ijerph15091970

**Published:** 2018-09-10

**Authors:** Saharuetai Jeamsripong, Rungtip Chuanchuen, Edward R. Atwill

**Affiliations:** 1 Research Unit in Microbial Food Safety and Antimicrobial Resistance, Department of Veterinary Public Health, Faculty of Veterinary Science, Chulalongkorn University, Pathumwan, Bangkok 10330, Thailand; chuanchuen.r@gmail.com; 2 Western Institute for Food Safety and Security, School of Veterinary Medicine, University of California, Davis, CA 95618, USA; ratwill@ucdavis.edu

**Keywords:** *Escherichia coli*, estuarine water, fecal coliforms, oyster, *Salmonella*, *Shigella*, *Vibrio parahaemolyticus*

## Abstract

This study characterized microbiological and chemical contamination of oyster meat and estuarine water in Phang Nga, Thailand. Pooled oyster meats (*n* = 144), estuarine waters (*n* = 96) and environmental parameters were collected from March, 2016 to February, 2017, and assessed for levels of total coliforms (TC), fecal coliforms (FC), *Escherichia coli* (EC), and *Vibrio parahaemolyticus* (VP), presence of *Salmonella* and *Shigella* and levels of heavy metals (Mn, Pb and Cd). The prevalence of TC, FC and EC were in 99.3%, 94.4% and 93.1% of oyster meat and 94.8%, 79.2%, and 78.1% of water, respectively. The average VP levels was 8.5 × 10^7^ most probable number (MPN)/g oyster. Prevalence of *Shigella* and *Salmonella* in the pooled oysters were 7.6% and 30.6%, respectively. The dominant *Salmonella* serovars were Paratyphi B followed by Seremban, and Kentucky. In contrast, the prevalence of *Shigella* were 27.1%, but *Salmonella* was not detected in estuarine water. Factors statistically associated with EC accumulation in oyster were level of FC, 7-day average precipitation, temperature, relative humidity, and presence of *Salmonella* in the sample. The optimal cutoff value of EC to predict *Salmonella* in oyster was 420 MPN/g. Results indicate this area has relatively safe levels of heavy metals, whereas bacterial contamination was very high for oysters.

## 1. Introduction

Shellfish products have been recognized as relatively inexpensive and nutritious sources of protein, minerals and vitamins, and there is growing global demand for shellfish products given the wide recognition of their nutritional value [1]. Global fisheries and aquaculture production was 158 million tons (MT) in 2012, of which aquatic animal capture was 91.3 MT compared to 66.6 MT from aquaculture. The export of fish and fishery products from Thailand has been ranked among the top 10 countries, with 6.5 MT in 2014 [2]. In Thailand, bivalve production was estimated at 210,000 tons in 2014 [2], with most of these seafood products consumed domestically due to the high consumer demand in the country. Despite this high domestic demand, aquaculture production of shellfish such as oysters is at risk from human activities like environmental pollution. Development of an aquaculture industry for oysters in Phang Nga Bay has benefited from a plentiful supply of natural oyster larvae, no excessive influx of fresh water, intact shorelines and an enclosed and protected growing area. *Saccostrea commercialis*, *Crassostrea lugubris* and *C. belcheri* are commercially significant oyster species in this region of Thailand, with the latter two species enjoying high commercial market value and commonly found in the south-west coast. 

Oyster farming in Thailand is generally located in shallow brackish or estuarine water close to the grower community. This proximity of the shellfish beds to the grower community heightens the risk of fecal-borne pathogen contamination of cultured oysters if community sanitation and sewage treatment processes are not fully adequate, especially during the rainy season. The oyster hanging method is generally applied for raising oysters in the southern Thailand. This method uses cement and nylon robe to attach two oysters together. Briefly, oyster shells are connected with cement, and a nylon rope is passed through the center of the shells to combine the oyster clutch. One rope contains approximately seven clusters of oysters.

Bivalves are effective filter feeders and readily concentrate biological contaminants including pathogenic and non-pathogenic bacteria and chemical pollutants from their environment. The accumulation of these contaminants in oysters poses a food safety hazard for humans. Numerous foodborne outbreaks of *Vibrio*, *Shigella*, *Salmonella*, *Clostridium botulinum* and *Staphylococcus aureus* have been linked to the consumption of raw or partly cooked oysters [3,4,5,6]. For example, *Salmonella* was detected in 7.4% of oysters from 36 bays throughout the United States (US) [7]. *Shigella* has been responsible for many seafood-associated outbreaks, mainly from consumption of raw oysters [8,9]. Contamination with *Vibrio parahaemolyticus* (VP) and *V. cholerae* in oysters have been frequently reported from different regions of the world, including the US, Italy, Chile, China, and Taiwan [6,10,11,12,13,14], leading to foodborne gastrointestinal infection.

Heavy metal contamination can occur in higher levels with mollusks and shellfish compared with other finfish products. The concentrations of heavy metals such as lead (Pb), cadmium (Cd), and zinc (Zn) were significantly higher in oysters than clams and mussels collected from Victoria harbor, southern China [15]. In the estuary of Lamnyong River in Indonesia, the levels of Pb, Cd, and Zn in oysters were higher than the maximum limit of oyster meat [16]. Even though, manganese (Mn) is an essential trace element used for nutrient absorption, metabolic enzyme function and bone development, the overconsumption of Mn can cause adverse effects on a neurological system such as cognitive psychiatric and Manganism, a disease like Parkinson’s in humans [17]. In general, oysters naturally grow in the estuarine area of brackish water, which possibly can receive polluting discharges from anthropogenic activities. The contamination of heavy metals can be easily accumulated in oyster meat. Monitoring of heavy metal contamination in oysters is needed to ensure shellfish safety for human consumption. Thus, mussels and oysters have been used as sentinels of heavy metal contamination [18,19,20], whereas fecal coliforms (FC) and *Escherichia. coli* (EC) serve as bio-indicators of seafood product contamination as well as proxies of environmental contamination of shellfish industrial production [21]. Even though these bacterial indicators are not directly pathogenic to humans, subgroups of EC such as *E. coli* O157:H7 and other shiga-toxin producing strains are proven foodborne hazards to human health. Thus, marine surveillance of bacterial indicators and pathogens, along with heavy metal accumulation should be conducted to monitor the distribution of these contaminants in shellfish in an effort to ensure safe seafood production.

In Thailand, shellfish aquaculture has grown to serve both domestic and international demand. At the same time, an increase in domestic and international tourist travel in southern Thailand, growth in local communities and industries in this region, and intensification of land use could potentially threaten aquaculture environment and estuarine water quality. To date there is limited data on environmental factors impacting on estuarine water quality and seafood contamination in southern Thailand. Hence, evaluation of baseline biological and chemical contamination of estuarine water quality and cultured shellfish is needed in order to prevent foodborne and waterborne diseases, especially for seafood consumed raw such as oysters. The novelty of this study is to explore potential contributing factors that could be used to quantify the number of bacterial contaminations of oyster meat and estuarine water, and to estimate an optimal cut-off value of EC contamination to predict *Salmonella* contamination of oyster meat. The objectives of this study were to: (1) measure bacterial accumulation of total coliform (TC), FC, EC, VP, *Salmonella*, and *Shigella* in cultured oysters and surroundings estuarine water; (2) quantify concentrations of Cd, Mn and Pb accumulated in oysters and estuarine waters from oyster aquaculture regions within Phang Nga Bay; (3) build statistical models to predict the association between biological and chemical contaminants in these bivalve ecosystems and potential environmental factors influencing oyster food safety; and (4) estimate the cutoff value of EC contamination among pooled oyster meat to predict *Salmonella* contamination of oyster.

## 2. Materials and Methods

### 2.1. Oyster Site Selection and Oyster and Estaurine Water Sample Collection

This study was conducted on oyster farms located in Marui canal at Phang Nga Bay along the Andaman Sea, southern Thailand between March 2016 and February 2017. These oyster sampling stations were selected based on the relatively new location of oyster aquaculture, and there is no string of regulations/guidelines to control oyster food safety and to select a proper oyster aquaculture area. The market for oysters from this area are directly serves many restaurants to Phang Nga, Krabi, and Phuket provinces, which are considered as famous tourist attractions, both nationally and internationally, in southern Thailand.

A total of 240 samples including fresh pooled oyster meats (*C. lugubris* and *C. belcheri*) (*n* = 144) and estuarine water (*n* = 96) were collected one year. Three pooled oyster samples along with two 500 mL samples of estuarine water were collected each month for 12 months from each of four different sampling locations. One pooled oyster meat sample contained 10–12 market-size oysters, which had been suspended at a depth of 1.5–2 m on a rope in the oyster production area. The field crews were trained on how to properly identify mature oysters that were close to marketable size, approximately 10–12 months of age. The estuarine water was aseptically collected into a sterile polyethylene bottle at 0.3–0.5 m below the water surface adjacent to the oyster collecting sites. All samples were stored at 10 °C during transportation and delivered to the laboratory within 24 h after collection.

### 2.2. Measurement of Environmental Factors

Ambient air temperature (°C), relative humidity (RH) (%), season (rainy: mid-May to early-October, winter: mid-October to early-February, summer; mid-February to early-May), the stage of tidal condition (flooding or ebbing tide), precipitation (yes or no at time of sampling), current wind speed (m/s), instantaneous wind speed (m/s), and average wind speed (m/s) were recorded by an air flow meter anemometer wind meter (Kestrel 3000, Nielsen-Kellerman, Boothwyn, PA, USA) monthly during each sampling event. In addition, 7-day averages of environmental parameters were calculated for wind speed (m/s), RH (%), precipitation (mm), and ambient air temperature (°C) by recording values every three h over a 7-day period and then summarized. These environmental parameters were included in this study which potentially influence on the bacterial and heavy metals contamination of oysters and estuarine waters. The weather monitoring data was retrieved from the Phang Nga weather station of the Thai meteorological department (https://tmd.go.th).

### 2.3. Determination of Total Coliforms (TC), Fecal Coliforms (FC) and Escherichia coli (EC)

All oyster meat and estuarine water samples were tested for TC, FC, EC, VP, *Salmonella*, *Shigella* and heavy metals. After samples were transported to the laboratory, the bacteriological and heavy metal assays were performed within 24 h after sample collection. All debris on oyster shells was first removed with a stiff scrub brush and the oyster shells were externally disinfected with alcohol 70% prior to shucking of the oyster meat with a sterile shucking knife. For oyster preparation, oyster meat (⁓300 g or 10–12 oysters) was pooled into a sterile container and blended at high speed for 30–60 s. These pooled oyster meat samples were subjected to further microbiological and chemical analyses.

TC, FC and EC concentrations were determined as the most probable number (MPN) for oyster meats and estuarine waters according to the United States of Food and Drug Administration (US-FDA)’s Bacteriological Analytical Manual (BAM) with slight modifications [22]. Briefly, a total of 50 g of blended oyster meat was added into a flask containing of 450 mL of sterile phosphate buffered saline (PBS) (Difco, Sparks, MD, USA) to yield a 1:10 dilution. The individual sample of oyster mixture and estuarine water samples were then diluted in lactose broth (Difco) containing with a Durham tube for a three-tube MPN method at least four dilutions (e.g., 10^−2^, 10^−3^, 10^−4^, and 10^−5^). The lactose broth tubes were incubated at 37 °C overnight. The production of gas in the Durham tubes was recorded as a positive at 24 h. From each lactose broth tube, one loopful of suspension was transferred to brilliant green lactose bile (BGLB) (Difco) and incubated at 35 °C for 24 h. The examination of gas production was observed at 48 h. For the FC confirmation test, a loopful of the lactose broth tubes from the previous step was transferred to a tube of EC broth (Difco), and incubated at 44.5 °C. The gas production was observed at 24 h. If no gas production, the tubes were incubated until 48 h. The results were used to calculate concentrations of FC. For confirmation of EC, a loopful of EC broth was streaked on Levine-eosin methylene blue (L-EMB) (Difco) agar plates and incubated overnight at 37 °C. Suspected EC colonies were flat and dark centered, with or without metallic sheen. Up to 5 suspicious colonies were transferred from L-EMB plates to plate count agar (PCA) (Difco), and incubated at 35 °C overnight. Positive EC colonies were confirmed biochemically by using Indole production. Suspected colonies were inoculated in tryptone broth (Difco) at 35 °C for 24 h. After the overnight incubation, 0.2–0.3 mL of Kovacs’ reagent was added. The present of cherry red color in the upper layer on was recorded as positive EC.

### 2.4. Determination of Vibrio Parahaemolyticus

The concentration of VP was determined in both oyster meat and estuarine water samples using US-FDA’s BAM method [23]. Aliquots of mixture solution of oyster meat or estuarine water samples from the previous step were serially diluted in triplicate with Alkaline Peptone Water (APW) (Difco) in at least three consecutive tubes (i.e., 10^−3^, 10^−4^, 10^−5^, 10^−6^, and 10^−7^) to determine the MPN of VP. The APW tubes were incubated at 37 °C for 24 h. A loopful of APW tubes from the top 1–1.5 cm of the three or four highest dilutions with positive (turbid tubes) were then streaked onto thiosulfate-citrate-bile salts-sucrose (TCBS) (Difco) and CHROMagar™ *Vibrio* (HiMedia Laboratories Ltd., Mumbai, India) agar plates. The TCBS and CHROMagar™ *Vibrio* plates were incubated at 37 °C overnight. Presumptive colonies were streaked onto tryptic soy agar (TSA) (Difco) plate supplemented with 2% NaCl. Positive colonies were then biochemically confirmed. On TCBS agar plates, positive colonies of VP were round, opaque and green colonies with 2–3 mm in diameter. On CHROMagar™ *Vibrio* plates, positive colonies were mauve color. Presumptive colonies of VP were plated on PCA (Difco) and incubated at 35 °C for 24 h. Oxidase test and arginine glucose slants (AGS) (Difco) were used to biochemically confirmed VP. Suspected VP colonies were re-streaked in TSA agar plates supplemented with 2% NaCl. The positive VP colonies were positive on oxidase test. In addition, suspected VP colonies were stabbed and streaked into AGS (Difco) and incubated at 37 °C overnight. The positive colonies of VP were purple in slant and yellow in butt without H_2_S gas production. Total VP was recorded in MPN/g oyster meat and MPN/100 mL in estuarine water.

### 2.5. Isolation of Salmonella *spp.*

The present of *Salmonella* was analyzed using the protocol as described by the US-FDA’s BAM method [24]. Twenty-five g of blended oyster meat was weighted from the pooled oyster preparation and mixed with 225 mL of lactose broth with vigorous shaking for 2–3 min. Estuarine water samples were mixed with double strength sterile lactose broth to receive 1:2 dilution. Both suspensions were set for 60 min at room temperature (25 °C) and then incubated at 37 °C for 24 h. Then 0.1 mL of each mixture solution was transferred into 10 mL of Rappaport–Vassiliadis (RV) medium (Difco) and incubated for 24 h at 42 °C. A loopful of individual suspension was streaked to xylose lysine deoxycholate (XLD) (Difco), MacConkey (Difco) and Hektoen enteric (HE) (Difco) agar plates. After overnight incubation at 37 °C, presumptive colonies of *Salmonella* spp. were observed pink color with or without black centers on XLD agar, colorless (lactose negative) on MacConkey agar, and blue colonies with or without black centers on HE agar plates. All suspected *Salmonella* colonies were then confirmed by biochemical test using triple sugar iron (TSI) (Difco) agar. TSI slant agar was incubated at 37 °C for 24 h. Observed red slant and yellow butt with gas production were recorded as positive *Salmonella*. One typical colony of *Salmonella*/positive sample was selected for serotyping using slide agglutination assay with specific antilipopolysaccharide antibodies based on the Kauffmann-White scheme method [25].

### 2.6. Isolation of Shigella *spp.*

*Shigella* was detected using the protocol described by US-FDA’s BAM methods with slight modification [26]. Twenty-five g of blended oyster meat and 25 mL of estuarine water samples from the original sample was weighted and enriched using 225 mL of *Shigella* and *Salmonella* broth (Difco). The suspension was held at room temperature at 25 °C for 10–15 min and then static incubated at 37 °C overnight. A loopful of suspension was streaked onto MacConkey agar (Difco) plate, and the plates were incubated at 37 °C for 24 h. The typical colonies of *Shigella* are small (2 mm of diameter), circular, smooth and transparent on MacConkey agar. All suspected colonies of *Shigella* spp. were streaked onto plate count (PCA) (Difco) agar for biochemical test using TSI slant agar. The observed red slant and yellow butt without H_2_S production were recorded as positive *Shigella* spp.

### 2.7. Determination of Heavy Metals

Levels of Mn, Pb and Cd in oyster meat and estuarine water were quantified using atomic absorption spectrophotometry (AAS: Varian model AA280FS, Agilent, Santa Clara, CA, USA) as described by Association of Analytical Communities (AOAC) international [27]. Briefly, five grams of blended oyster meat was dried at 70 °C and the residues were added to 10 mL of 65% HNO_3_ (Suprapure^®^ grade, Merck, Washington, DC, USA) and set at room temperature (25 °C) overnight. The mixture suspension was dried on a hot plate at 50 °C to receive approximately 1 mL of residues. Distilled water was added to the residue until it achieved 25 mL of volume. Estuarine waters (~200 mL) were mixed and filtered passing through 11 µm of membrane filter (Whatman, Maidstone, UK), which filtered large particles. Then, the suspension was re-filtered with a 0.4 µm membrane filler (Whatman). Thirty mL of HNO_3_ were added into the filtered suspension and set overnight at room temperature. The mixture solution was dried at 50 °C. Twenty-five mL of distilled water was added into the solution and filtered through 0.45 µm membrane filter (Whatman). All samples were stored at 4 °C and submitted to heavy metal analysis within 24 h at the Science and Technology Research Equipment Centre (STRE), Chulalongkorn University.

### 2.8. Statistical Analyses

The levels of TC, FC, EC, and VP in cultivated oysters and estuarine waters were estimated by using a MPN calculator available elsewhere. Negative binomial regression was used to test the association between levels of EC in oysters (MPN/g) or estuarine waters (MPN/100 mL) and daily and 7-day environmental factors: average wind speed (m/s), instantaneous wind speed (m/s), ambient air temperature (°C), RH (%), precipitation (mm), the stage of tidal condition (flooding or ebbing tide), season (rainy, winter, summer), month when sampling occurred, concentrations of TC, FC and VP, presence of *Salmonella* and *Shigella* in the sample, and heavy metal levels (ppm). Logistic regression and receiver operating characteristic (ROC) analyses were performed to test the association between the predictive ability of various covariates such as environmental factors and levels of EC and the presence of *Salmonella* in oyster meat. Regarding ROC analyses, the index of accuracy and Youden index were used to determine the optimal cutoff value for levels of EC to predict *Salmonella* in oysters [28,29]. Given that oysters were collected/month at each location for 12 months, we adjusted the *p*-values and CI’s of regression analyses for potential correlated data within location for EC levels using a robust variance estimator. Univariate regression models for all independent variables were first screened for potential significance. Using the *p*-value for initial inclusion, a backward stepping algorithm was used to build the multivariable mixed-effects negative binomial regression and logistic regression models for oysters and estuarine waters, with a *p*-value ≤ 0.05 based on a likelihood ratio test for retention in the final model. Statistical analysis was performed using Stata version 14.0 (StataCorp, College Station, TX, USA). For statistical inferences, two-sided hypothesis tests were used with a 5% significance level.

## 3. Results

### 3.1. Sampling Location and Environmental Condition

**Figure 1:**
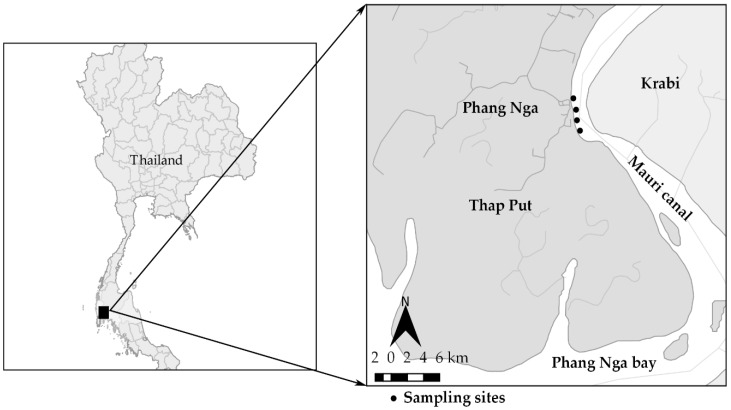
Four sampling sites are indicated as black circle dots, where pooled oyster and estuarine water samples were collected from Marui cannal in Phang Nga bay from southern Thailand. Source: Map of Thailand (www.diva-gis.org/gdata).

The pooled oyster meat and estuarine water samples were collected each month from four different oyster locations in Thap Put district (Latitude 8.541530 and Longitude 98.638060 or degree 8°32′29.508′′ N and 98°38′17.016′′ E) at Phang Nga Bay along the Andaman Sea in southern Thailand (Figure 1). The map was created by the QGIS program version 2.18 (Las Palma de Gran Canaria, Spain). Environmental samples were measured daily by an air flow meter anemometer wind meter on sampling day, and average 7-day parameters prior to sampling were retrieved from the Thai meteorological department. Wind parameters averaged over the 12 months from March 2016 to February 2017 were 1.22 ± 0.89 m/s for current wind speed, 1.67 ± 1.09 m/s for instantaneous wind speed, and 0.91 ± 0.57 m/s for average wind speed. The highest current wind spend (4.05 m/s), instantaneous wind speed (5.08 m/s), and average wind speed (2.73 m/s) were observed during winter, while the lowest current wind speed (0.53 m/s), instantaneous wind speed (0.68 m/s), and average wind speed (0.53 m/s) were reported during the summer. Temperature fluctuated between 28.9 °C in winter and 34.9 °C in summer with an average temperature at 31.6 °C, typical of tropical coastal ecosystems in SE Asia. The average RH was 73.7% and ranged from 49.5–88.7%. Precipitation was limited to six months in May, June, August, and September through November in 2016.

### 3.2. Concentrations of Total Coliforms, Fecal Coliforms, and E. coli

In pooled oyster meat samples (*n* = 144), the observed prevalence of TC, FC, and EC was 99.3%, 94.4%, and 93.1%, respectively. The average concentrations and standard deviation of these bacteria in oyster and estuarine water samples are shown in Table 1. The range of bacterial concentrations were 0.6–3.2 × 10^4^ MPN TC/g, 0.6–2.2 × 10^4^ MPN FC/g and 4.6–2.2 × 10^4^ MPN EC/g oyster meat. In estuarine water, the prevalence of TC, FC, and EC (*n* = 96) was 94.8%, 79.2%, and 78.1%, respectively. Bacterial concentrations ranged from 3.3–1.1 × 10^4^ MPN TC/100 mL, 8.0–1.1 × 10^4^ MPN FC/100 mL, and 8.0–4.6 × 10^3^ MPN EC/100 mL, respectively.

### 3.3. Concentrations of V. parahaemolyticus

All oyster and estuarine water samples were positive of VP, with high concentrations of VP in oysters observed throughout the year with an average concentration of 8.5 × 10^7^ (±3.8 × 10^6^) MPN/g of oyster meat. The highest concentration of 1.1 × 10^8^ MPN VP/g oyster was reported during the rainy to winter seasons. In estuarine water, the average concentration of VP was 3.8 × 10^5^ (±4.7 × 10^4^) MPN/100 mL (Table 1), with highest concentration of 1.1 × 10^6^ MPN/100 mL observed in winter, while the lowest VP level was 1.1 × 10^4^ MPN/100 mL in summer.

### 3.4. Presence of Salmonella *spp.* and Shigella *spp.*

**Figure 2:**
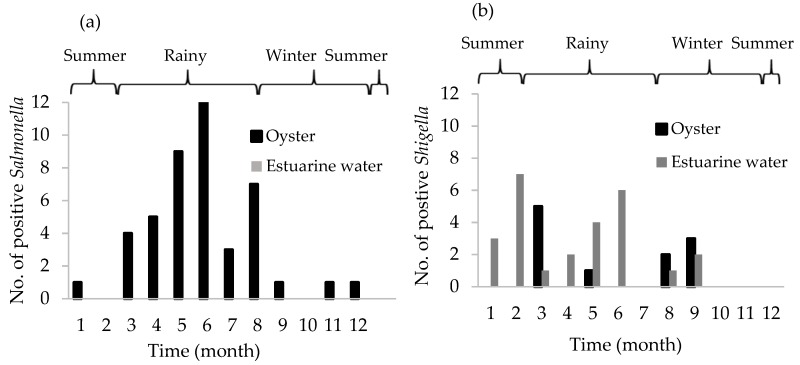
Number of positive samples for *Salmonella* spp. (*n* = 44/144) and Shigella spp. (*n* = 11/144): (**a**) The number of positive *Salmonella* samples; (**b**) The number of postitive *Shigella* samples of pooled oysters and estuarine water from Phang Nga Bay, southern Thailand. Sample collection started from March 2016 (month 1) to February 2017 (month 12).

The positive *Salmonella* of pooled oysters and estuarine water samples were presented in Figure 2. In oysters, the prevalence of *Shigella* (*n* = 11) and *Salmonella* (*n* = 44) was 7.6% and 30.6%, respectively. Among the eleven positive *Shigella* samples, the majority occurred in May (*n* = 5) and November (*n* = 4). Among the 44 *Salmonella* positive samples, the majority occurred during the rainy season in August (*n* = 12), followed by July (*n* = 9) and October (*n* = 7). The predominant *Salmonella* serovars were Paratyphi B (22.7%), Seremban (11.4%), and Kentucky (9.1%) presented in Table 2. For estuarine water samples, the prevalence of *Shigella* was 27.1% (*n* = 26), with the majority of positives in April (*n* = 7) and August (*n* = 6). All estuarine water samples tested negative for *Salmonella* spp.

### 3.5. Heavy Metal Concentrations

**Figure 3:**
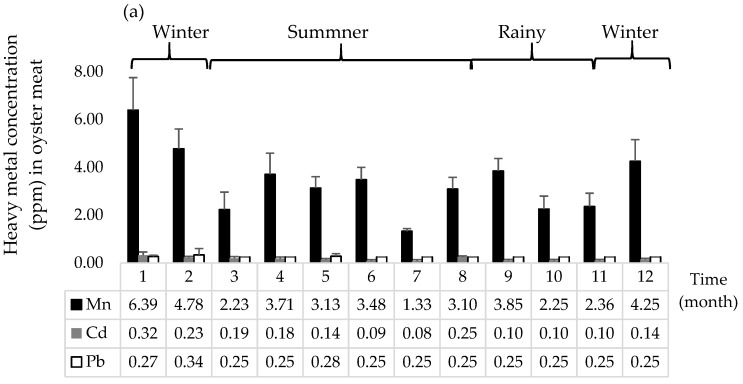

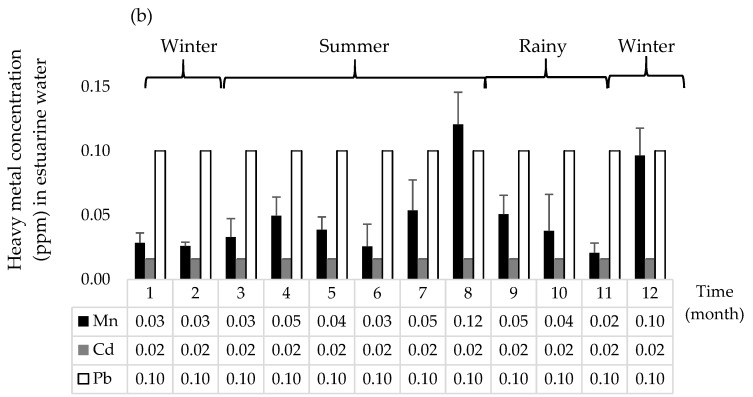
Heavy metal concentration (ppm) of manganese (Mn), cadmium (Cd), and lead (Pb): (**a**) oyster meat samples; (**b**) estuarine waters from Phang Nga Bay, southern Thailand. Samples were collected from March 2016 (month 1) to February 2017 (month 12).

Average concentrations and standard deviation of Mn, Cd, and Pb in pooled oyster meat were 3.41 (±0.12), 0.16 (±0.01) and 0.26 (±0.01) ppm, respectively (Figure 3), which was much higher compared to estuarine water samples which had levels of Mn of 0.05 (±0.0) ppm and below the detection limit of 0.01–0.02 ppm for Pb and Cd, respectively. The highest metal contamination in pooled oyster meat occurred in March for Mn (6.39 ppm) and Cd (0.32 ppm) and in April for Pb (0.34 ppm).

### 3.6. Multivariable Mixed-Effects Negative Binomial Regression Model

**Figure 4:**
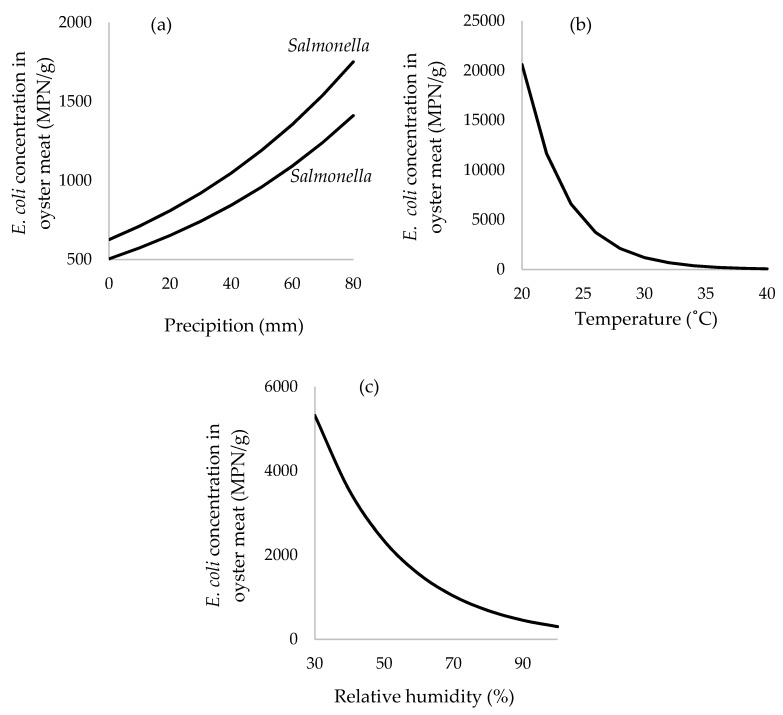
Predicted average concentration of EC (MPN/g of oyster meat) as a function of: (**a**) average precipitation over 7 days stratified by the presence of *Salmonella* spp.; (**b**) temperature prior to sampling; (**c**) relative humidity (%). Note: (**b**,**c**) were predicted based on the presence of *Salmonella* in the oyster sample.

**Figure 5:**
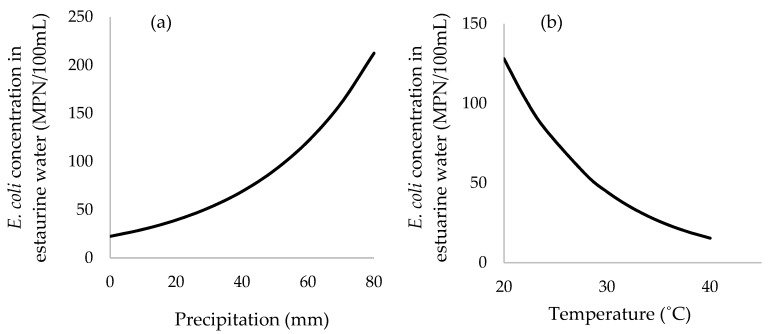
Predicted average concentration of EC (MPN/100 mL of estuarine water) as a function of: (**a**) average precipitation over 7 days; (**b**) ambient air temperature

Average precipitation during the previous 7 days, ambient air temperature, relative humidity, concentration of FC, and the presence of *Salmonella* in the sample were all significant parameters associated with the concentration of EC in oysters (Table 3, Figure 4). For estuarine water, average precipitation during the previous 7 days, air temperature and concentration of FC were significantly associated with EC (Table 4, Figure 5). Interestingly, average 7-day precipitation and air temperature were positively and negatively associated with EC in both predictive models of oysters and estuarine waters. This suggested that the same mechanism was observed in oysters and estuarine waters.

### 3.7. Logistic Regression and Receiver Operating Characteristic (ROC) Curve Analyses

**Figure 6:**
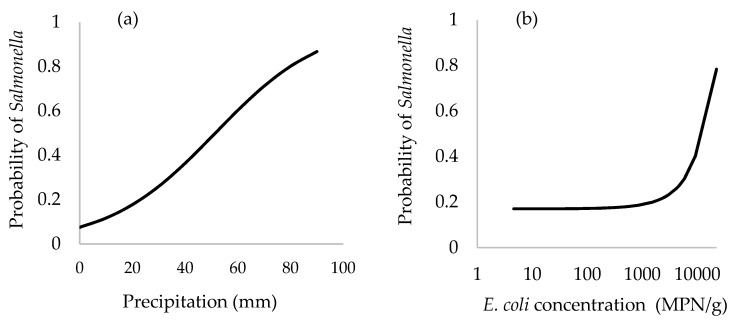
Predicted probability of detecting *Salmonella* in pooled oyster samples as a function of: (**a**) average precipitation during previous 7 days; (**b**) the levels of EC (MPN/g oyster meat).

**Figure 7:**
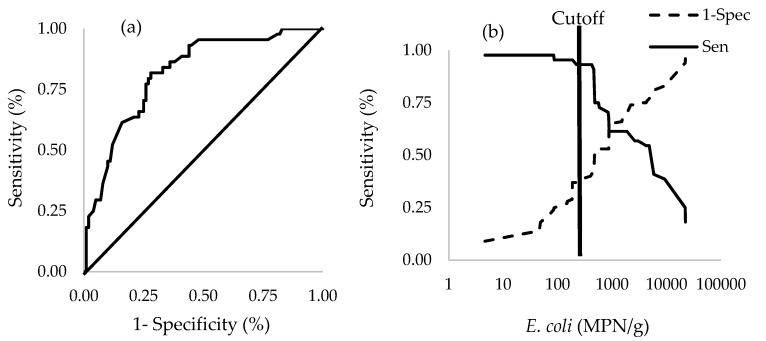
Accuracy for using EC to predict *Salmonella* contamination in pooled oyster samples: (**a**) ROC curve; (**b**) sensitivity and specificity for using EC concentration (MPN/g oyster meat) to predict *Salmonella* contamination in oysters, with a vertical line indicating the optimal cutoff value of 420 EC (MPN/g) based on the Youden index.

Average 7 days of precipitation, concentration of EC, and an interaction term between EC levels and average precipitation prior 7 days were significantly associated with the presence of *Salmonella* in oyster meat (Table 5, Figure 6). The area under the ROC curve for this logistic regression model shown in Table 5 was 81.8% (CI = 74.5–89.0%) (Figure 7a), which was significantly greater (*p* < 0.0001) than the null value of a non-informative test (AUC = 0.5). With respect to the ability of this logistic regression model to predict the presence of *Salmonella* in oyster meat, the optimal cutoff value for EC concentration was 420 MPN/g of oyster which generated a test sensitivity of 93.2% and test specificity of 41.0% (Figure 7b).

## 4. Discussion

Average concentrations of indicator bacteria observed in oysters from Phang Nga Bay, southern Thailand, ranged from 10^3^–10^4^ CFU/g oyster meat. Previous work has found FC levels in oysters to range from 1–10^3^ MPN/g meat [30,31]. These levels of bacterial contamination can be contrasted against regulations for maximum bacterial levels in shellfish for human consumption. For example, the European Union (EU) has criteria for class A (<230 EC or <300 FC in 100 g of shellfish meat) for human consumption without further processing and for class B (<4600 EC in 100 g of oyster) for additional depuration, relaying, or heat treatment, while the Codex Alimentarius standard indicates <700 EC in 100 g of shellfish meat [32,33]. Given these standards, about 90% of oyster samples from Phang Nga Bay would exceed the international trade standard of Codex Alimentarius for human consumption. According to US-FDA, the National Shellfish Sanitation Program (NSSP) sets a criteria of <14 FC/100 mL of seawater for shellfish growing areas [34]. In the Phang Nga Bay, the concentration of FC observed in estuarine water was greater than the NSSP guideline for 78% of the samples over a 12 month period. This suggests that the growing conditions and harvestable oysters from this region of southern Thailand are potentially of low sanitation, indicating potential risks for human consumption without additional steps to improve seafood safety, such as adequate depuration and thorough cooking to reduce the load of bacteria in oysters.

It is important to note that the valid assumption is hold when the observed ranges of parameters used in the EC predictive model. On the assumption that the regression model for EC levels in oyster meat is valid (Figure 4), the parameters of such average precipitation and FC levels in oyster meat may provide a predictive model or early warning system for oyster food safety. For example, for each additional increase of 100 FC in oyster meat, levels of EC increase by ~1.015-fold (or 1.5%, e^(0.000149×100)^ = 1.015). Monitoring FC levels rather than EC might function as a reliable indicator of shellfish quality and further reduce costs of surveillance due to cheaper assays compared to EC analysis [20,35]. Levels of EC in oysters varied substantially with the amount of average precipitation during the previous 7 days. Specifically, for each additional 10 mm of precipitation during the previous 7 days, levels of EC increased in oyster meats on average by ~1.14-fold (or 14%, e^(0.0128×10)^ = 1.137) or about 0.06 log. This finding is consistent with previous studies that found precipitation was a significant predictor for concentrations of EC in oysters. For example, EC levels were 8.7 × 10^3^/100 g of shellfish following rainfall events with positive correlation with the levels of EC in estuarine water [36]. In India, fecal indicators were higher during the monsoon season from June to September compared to either the pre- or post-monsoon season [32]. From a linear mixed-model that predicted EC contamination on oysters (Figure 4), every increase of 10 mm of precipitation, the EC concentration in oyster flesh increases 1.14 fold (e^0.0128×10^). The mechanisms explaining this observation of higher levels of EC in oyster meats during periods of higher precipitation could be the result of higher levels of sewage and bacterial pollution draining into the shellfish growing areas, along with higher river or stream flow regimes increasing local turbulence and resuspension of bacterial-laden sediments [37]. This suggests that with additional data regarding linkages between specific patterns of 7-day average precipitation and subsequent EC levels in oysters, thresholds could be established for maximum allowable amounts of 7-day precipitation above which shellfish harvesting would not be allowed without additional interventions, such as requiring oysters to depurate for specified amounts of time and/or disallowing the consumption of raw oysters when harvested within several days following a rainfall exceedance.

The determination of *Salmonella* and *Shigella* in terms of presence or absence is used to explain oyster safety for human consumption. The 30.6% prevalence of *Salmonella* in oyster meat measured in this study was generally higher than has been observed in earlier studies. For example, *Salmonella* varied from 1.3–8% in shellfish for both domestic and export seafood products in the U.S. [30]. Interestingly, higher levels of EC were observed when *Salmonella* was present in the oysters (Figure 4a). When 7-day precipitation was at 40 mm, predicted levels of EC in oysters was 6.2 × 10^3^ MPN/g when *Salmonella* was present and only 2.3 × 10^3^ MPN/g when *Salmonella* was absent, or about 2.7-fold higher. This association may be driven in part by conditions of heavy precipitation leading to high rates of terrestrial runoff and other causes of waterborne bacterial pollution during storm flow conditions, resulting in both EC and pathogenic bacteria like *Salmonella* substantially increasing in oysters. More critically, the significance of *Salmonella* in and above 7-day precipitation suggests there is an additional mechanism of EC pollution for the shellfish-growing areas of Phang Nga Bay in southern Thailand, and when this unknown mechanism is operating it leads to both *Salmonella* contamination of oysters in addition to a 2.7-fold higher level of EC in oysters. It may be a productive area of future inquiry to conduct careful traceback and/or molecular studies of waterborne *Salmonella* in the local watershed to discover this currently unknown mechanism, which if rectified might not only reduce EC levels in oysters but also reduce *Salmonella* contamination. Nonetheless, detection of *Salmonella* may not consistently correlate with concentrations of indicator bacteria [38], whereas Hood et al. suggested that the concentration of FC could be used to estimate the number of pathogenic bacteria such as *Salmonella* [20]. In this study, the main serovar isolated from raw cultivated oysters has been found to be *Salmonella* Paratyphi B; this pathogen is the third most common serovar found in seafood products causing foodborne disease outbreaks according to the US-FDA (35). In the U.S., sources of *Salmonella* Paratyphi B isolated from the aquatic environment have been reported in the Little River and Oconee River Basins and other watersheds [39,40].

Both RH and ambient air temperature were negatively associated with higher levels of EC in oyster meat (Figure 4). The seasons that correlate with high RH and high air temperature are during southern Thailand’s summer (Feb–May) and rainy (May–October) season. These observations are consistent with the findings from Kerala, India, where there was strong negative correlation (R^2^ = −0.7) between the EC levels and seawater temperature within a range of temperatures from 26–32 °C [35]. One mechanism to explain these findings is that a main inactivating factor for EC survival in brackish waters was sunlight [41], which tends to be high during summer. A low level of FC in shellfish was observed during the colder winter, with the concentration of EC influenced by seasonal salinity, turbidity, and solar radiation [42]. Furthermore, higher air temperatures tend to occur during Thailand’s seasonal dry period, thereby eliminating EC contamination in oysters that is driven by precipitation events. The finding is supported by the observation that the survival of indicator bacteria is negatively associated with temperature [43,44,45]. In contrast, increased levels of *Vibrio* occurred during higher temperatures [46]. Based on the negative coefficient from the negative binomial model in Table 3, a 10% increase in RH was associated with a 0.66-fold decrease (e^−0.041×10^ = 0.66) in the concentration of EC in oysters. Similarly, for every additional one degree (°C) of ambient air temperature there was an associated 0.76-fold (e^−0.28×1^ = 0.76) reduction (~0.12 log reduction) in EC levels in oysters. Seasonal effects, such as air temperature and RH, appear to have strong associations with EC contamination of the oysters of Phang Nga Bay in southern Thailand.

Average air temperature and mean precipitation during the previous 7 days were negatively and positively associated with the levels of EC in estuarine water samples, respectively (Figure 5). Interestingly, the direction of these associations were the same as observed on the oyster model. For example, an additional one degree (°C) in ambient air temperature is associated with a 0.66-fold decrease (e^−0.11×1^ = 0.90) in the concentration of EC in estuarine water, while each additional 10 mm in cumulative precipitation during the previous 7 days was associated with a ~1.32-fold (or 132%, e^0.028×10^ = 1.32) in the levels of EC. This suggests that early monitoring of ambient air temperature and 7-day cumulative precipitation could be used to predict the quality of oyster meat and estuarine water.

Factors associated with the presence of *Salmonella* in oyster meat were levels of EC and average precipitation during the previous 7 days (Table 3, Figure 6). Average 7-day precipitation was also a significant factor associated with the concentration of EC in oyster meat and estuarine water. Assuming the logistic regression model is valid, the levels of EC in oyster meat could be used as a predictor for *Salmonella* spp. in oyster meat. This finding is supported by Hood’s finding that levels of indicator bacteria could be used to estimate pathogenic bacteria contamination in oyster samples [21]. The area under the ROC curve was 82% for using EC levels to predict *Salmonella* contamination in oyster meat. Moreover, based on the Youden index (maximizing the difference between sensitivity and 1-specificity under the ROC curve), the optimal cutoff value of 420 EC MPN/g of oyster meat when used to predict *Salmonella* contamination generated a test sensitivity of 93.2% and test specificity of 41.0%. These results indicate that once the concentration of EC was greater than 420 MPN/g oyster meat, there was a high probability of *Salmonella* contamination in oyster meat in southern Thailand. In contrast, when EC was less than 420 MPN/g oyster meat (test negative), there was a near equal chance for a true negative test (40.1% chance *Salmonella* is absent) or false negative test (59.9% chance *Salmonella* present) for *Salmonella*.

In this study, levels of Pb and Cd contamination in oysters did not exceed the limit proposed by Codex Alimentarius [33], indicating that oysters and estuarine water in the oyster growing area of the Phang Nga Bay are safe from heavy metal pollution. In our study, the concentrations of Cd and Pb in oyster meat were low which is similar to the studies in Malaysia and Iran. In the east coast peninsular of Malaysia, the low concentrations of Pb (0.17 ± 0.15 ppm) and Cd (1.60 ± 0.28 ppm) have been observed in oysters (*C. iredalei*) [47]. The concentrations of Cd and Pb were in acceptable limits, while the concentration of Zn, cooper (Cu), nickel (Ni), Pb, Chromium (Cr) and arsenic (As) were highly observed in oyster meat collected from the Persian Gulf in Iran [48]. However, the concentrations of Pb, Cd and Zn had exceeded the maximum limit for oyster consumption in Banda Aceh city, Indonesia [16]. Likewise, concentrations of Cd and Cr observed in oysters exceeded the limit for human consumption as reported in China [15]. Mn is currently considered an emerging contaminant, since it is a threat to human and environmental health [49]. The contamination of dissolved Mn was reported in seawater ranging from 10 to >10,000 ppm [50], which is much higher than the average of Mn concentration observed in oysters and estuarine waters in this study. The inconsistency of heavy metal concentrations have been observed, because the levels of contamination is closely relevant to the anthropogenic activities. Thus, monitoring of heavy metal contamination should be implemented to ensure that the harvested aquatic animals are safe for consumption.

## 5. Conclusions

This study characterizes the current conditions of bacterial and heavy metal contamination in the cultured oysters and estuarine water quality of southern Thailand. Significant associations were identified between environmental predictors such ambient air temperature and precipitation and the occurrence of EC and *Salmonella* and in oysters and estuarine waters of Phang Nga Bay in southern Thailand. Further epidemiological investigation of the impact on sanitary practices should be performed on polluted and unpolluted areas to enhance oyster safety. Assessment of environmental and biological parameter chaining can be performed prior to collecting oysters to avoid the bacterial contamination in oyster meat for human consumption. These findings could be used as a basis to develop a rapid warning system based on environmental parameters to predict levels of bacterial contamination in oysters in order to reduce the risk of foodborne illness associated with consumption of oysters. In conjunction with an early warning system, other factors such as increasing sanitary practices, consumption of adequately cooked seafood in proper time and temperature, and avoidance of harvesting oysters during heavy precipitation could function to collectively reduce the risk of bacterial contamination of oysters and enhance the safety of Thai seafood.

## Figures and Tables

**Table 1:** The average concentration and standard deviation (MPN/g of oyster meat or MPN/100 mL of estuarine water) of TC, FC, EC, and VP in pooled cultivated oysters (*n* = 144) and estuarine waters (*n* = 96) of Phang Nga Bay, southern Thailand, March 2016 to February 2017.

Sample	Bacterial Concentration ^1^			
	TC	FC	EC	VP
Oyster meat (MPN/g oyster)	9.31 × 103 (9.95 × 103)	6.43 × 103 (8.82 × 103)	4.58 × 103 (7.07 × 103)	8.50 × 107 (3.78 × 106)
Estuarine water (MPN/100 mL)	4.74 × 102 (1.37 × 103)	4.18 × 102 (1.33 × 103)	2.17 × 102 (7.06 × 102)	3.78 × 105 (4.70 × 104)

^1^ Arithmetic mean (standard deviation) of bacterial concentration; TC = total coliforms; FC = fecal coliforms; EC = *Escherichia coli*; VP = *Vibrio parahaemolyticus*.

**Table 2:** *Salmonella* serovars of pooled cultivated oysters (*n* = 44) of Phang Nga Bay in southern Thailand, March 2016 to February 2017.

Serovars	*n* (%)
Augustenborg	2 (4.55)
Austin	1 (2.27)
Braenderup	2 (4.55)
Colorado	2 (4.55)
Eastbourne	3 (6.82)
Enteritidis	1 (2.27)
Friedenau	2 (4.55)
II	1 (2.27)
IIIa	1 (2.27)
Kentucky	4 (9.09)
Larochelle	2 (4.55)
Limete	1 (2.27)
Lomita	1 (2.27)
Ndolo	3 (6.82)
Nola	1 (2.27)
Paratyphi B	10 (22.73)
Sao	2 (4.55)
Seremban	5 (11.36)
Total	44 (00.0)

**Table 3:** Mixed-effects negative binomial regression model for factors associated with the concentration of *E. coli* in pooled oysters from Phang Nga Bay, southern Thailand, March 2016 to February 2017.

Factors	Coefficient	95% CI ^1^	*p*-Value ^1^
Intercept	18.07	9.47 to 26.67	<0.0001
Concentration of FC ^2^	1.49 × 10^−4^	1.28 × 10^−4^ to 1.70 × 10^−2^	<0.0001
Average precipitation prior 7 days (mm)	1.28 × 10^−2^	4.75 × 10^−3^ to 2.09 × 10^−4^	0.002
Average temperature (°C)	−0.28	−0.50 to −0.065	0.011
Relative humidity (%)	−0.041	0.065 to −0.017	0.001
*Salmonella* present in sample			
No	0.0	-	
Yes	0.22	0.068 to 0.36	0.004

Akaike Information Criterion (AIC)= 2402.58. ^1^ The 95% confidence interval (CI) and *p*-values were adjusted for potential intra-group correlation within four oyster sampling locations. ^2^ FC = fecal coliforms (MPN/g of pooled oyster meat).

**Table 4:** Mixed-effects negative binomial regression model for factors associated with the concentration of *E. coli* in estuarine water from Phang Nga Bay, southern Thailand, March 2016 to February 2017.

Factors	Coefficient	95% CI ^1^	*p*-Value ^1^
Intercept	6.40	3.30 to 9.49	<0.0001
Concentration of FC ^2^	8.75 × 10^−4^	5.89 × 10^−4^ to 1.16 × 10^−3^	<0.0001
Average precipitation prior 7 days (mm)	0.028	0.023 to 0.033	<0.0001
Average temperature (°C)	−0.11	−0.20 to −0.01	0.030

Akaike Information Criteria (AIC) = 981.05. ^1^ The 95% confidence interval (CI) and *p*-values were adjusted for potential intra-group correlation within oyster sampling locations; ^2^ FC = fecal coliforms (MPN/100 mL of estuarine water).

**Table 5:** Logistic regression model for factors associated with *Salmonella* in pooled oyster samples from Phang Nga Bay, southern Thailand, March 2016 to February 2017.

Factors	Coefficient	95% CI ^1^	*p*-Value ^1^
Intercept	−2.52	−2.78 to −2.26	<0.0001
Concentration of EC ^2^	2.21 × 10^−4^	1.30 × 10^−4^ to 3.13 × 10^−4^	<0.0001
Average precipitation prior 7 days (mm)	0.049	0.031 to 0.068	<0.0001
Concentration of EC × precipitation	4.8 × 10^−6^	−6.12 × 10^−6^ to −3.48 × 10^−6^	<0.0001

Akaike Information Criteria (AIC) = 145.09. ^1^ The 95% confidence interval (CI) and *p*-values were adjusted for potential intra-group correlation within oyster sampling locations; ^2^ EC *= Escherichia coli* (MPN/g of pooled oyster meat).
